# Incidence and predictive factors of non adherence to therapy in young adults attending a psycho social center in Milan: a retrospective observational “Real world” study

**DOI:** 10.1192/j.eurpsy.2022.1779

**Published:** 2022-09-01

**Authors:** S. Vanzetto, G. Cirnigliaro, E. Piccoli, S. Dagoberti, M. Vismara, B. Benatti, C. Viganò, B. Dell’Osso

**Affiliations:** 1 Luigi Sacco University Hospital, Psychiatry 2 Unit, ASST FBF-Sacco, University Of Milan, Milan, Italy; 2 “Luigi Sacco” Department of medicine and surgery, University Of Milan, Milan, Italy; 3 “Aldo Ravelli” Center for Nanotechnology and Neurostimulation”, University Of Milan, Milan, Italy; 4 Department of Psychiatry and Behavioral Sciences, Stanford University, Milan, Italy; 5 “Centro per lo studio dei meccanismi molecolari alla base delle patologie neuro-psico-geriatriche”, University Of Milan, Milan, Italy

**Keywords:** Adherence to treatment, Promotion of mental health, Territorial psychiatry, young adults

## Abstract

**Introduction:**

Non adherence to psychotropic drugs is associated with negative outcomes, including hospitalizations, aggressive behaviors, suicide attempts and increased premature mortality. It represents a psychiatric challenge, especially in young adults who show higher risk of non-adherence to treatment

**Objectives:**

Firstly this study evaluates the incidence of non-adherence to therapy in 18-24 years patients from a Psycho-Social Center in Milan; and then to analyze the predictive factors of non-adherence.

**Methods:**

In this retrospective “Real Life” observational study, 120 outpatients aged 18 to 24 years, from Psycho-social Center of L. Sacco University Hospital in Milan, were recruited in 2019. Non-adherence to treatment, according to the World Health Organization, was considered “a modality of assuming medications that does not correspond to healthcare professionals’ recommendations”. Statistical analysis were performed with chi-square, ANOVA and linear regression tests, setting significance to p<0.05.

**Results:**

88 of 120 outpatients (73.3%) received an indication to psychopharmacological treatment. Of these, 23 (26.1%) did not show adherence to therapy. Results showed a positive association between non-adherence and increased hospitalizations (p <.01), oral antipsychotics (p<.05) and drop-out rates (p<.001). A significant correlation was also observed between non-adherence and Intellectual Disability (p<.05), Bipolar Disorder (p<.05), psychotic symptoms (p<.05), alterations in affectivity and mood (p<.005), alterations in sleep pattern (p<.05), school dropout (p<.05) and poor family support (p<.01).

**Conclusions:**

This study confirms that non-adherence has a relevant incidence in young-adults psychiatric population, highlighting the importance of effective and structured assessment in clinical practice to identify predictive factors and risk profiles associated with this phenomenon.

**Disclosure:**

No significant relationships.

